# Milk Quality and Carbon Footprint Indicators of Dairy Sheep Farms Depend on Grazing Level and Identify the Different Management Systems

**DOI:** 10.3390/ani11051426

**Published:** 2021-05-16

**Authors:** Javier Plaza, Isabel Revilla, Jaime Nieto, Cristina Hidalgo, Mario Sánchez-García, Carlos Palacios

**Affiliations:** 1 Area of Animal Production, Faculty of Environmental and Agrarian Sciences, University of Salamanca, Avenida Filiberto Villalobos 119-129, 37007 Salamanca, Spain; pmjavier@usal.es (J.P.); jaimenl@usal.es (J.N.); mariosancia@gmail.com (M.S.-G.); 2 Area of Food Technology, University of Salamanca, E.P.S. of Zamora, Avenida Requejo 33, 49022 Zamora, Spain; irevilla@usal.es; 3 Faculty of Economic and Business Science, University of León, Campus de Vegazana s/n, 24071 León, Spain; cristina.hidalgo@unileon.es

**Keywords:** grazing level, dairy sheep, carbon footprint, milk quality, multivariate techniques, management systems

## Abstract

**Simple Summary:**

In order to assess the effect of grazing level on milk quality and indicators related to the carbon footprint of dairy sheep farms, monthly data collection was carried out for 1 year on 17 farms in the region of Castilla y León (Spain). These data were analysed using a multivariate statistical procedure that allowed the association of the mentioned indicators with the grazing level and identifying the management system of the farms. It was shown that farms with higher grazing levels were more environmentally sustainable, as indirect gas emissions and energy consumption were much lower. Milk quality from these farms was higher in terms of total protein, fat, omega 3 fatty acids, conjugated linoleic acid and α-tocopherol levels.

**Abstract:**

Currently, there are very few studies in the dairy sheep sector associating milk quality and indicators regarding carbon footprint and their link to grazing levels. For 1 year, monthly milk samples and records related to environmental emissions and management systems were collected through surveys from 17 dairy sheep farms in the region of Castilla y León (Spain), in order to relate this information to the use of natural pastures under free grazing. Indicators were constructed on the collected data and subjected to a multivariate statistical procedure that involved a factor analysis, a cluster analysis and a population canonical analysis. By applying multivariate statistical techniques on milk quality and carbon footprint indicators, it was possible to identify the management system of the farms. From an environmental point of view, farms with a higher grazing level (cluster 4) were more sustainable, as they had the lowest carbon footprint (lower CO_2_, N_2_O and CO_2_ equivalent emissions per sheep and year) and the lowest energy consumption levels, which were gradually lower than those of farms in cluster 3; both indicators were much lower than those of farms in clusters 1 and 2. The milk quality of cluster 1 and 2 farms was significantly lower in terms of total protein and fat content, dry extract, omega-3 fatty acid levels and α-tocopherol content than farms in clusters 3 and 4, which had higher accessibility to grazing resources. In sum, the higher the use of natural resources, the lower the external inputs the farms required and the lower environmental impact and energy costs they have.

## 1. Introduction

In the Spanish dairy sheep sector, there are different management systems, from mainly extensive systems that base sheep feeding only on grazing, to very intensive systems where feed complementation prevails with little or no grazing practice [1]. In this context, several studies have been conducted comparing the composition and quality of the products according to the different farming systems [2,3]. However, there is limited scientific evidence of the relationship between the quality of products and the different farming systems with the main carbon footprint indicators. Thus, the great efficiency of small ruminants in transforming forage products of limited nutritional quality into animal products of high physicochemical and organoleptic quality, with a very low fossil fuel consumption, is being underestimated [4].

Of all the productive factors that constitute the management system, feeding is considered to be the most important in the quality of both meat and milk products. In fact, the feeding system chosen by the farmer can be quickly assessed by the product quality [5]. Extensive systems are the ones that best fit the usual behaviour patterns of these small ruminants, and their practices make the milk acquire specific organoleptic and physicochemical characteristics, such as an increase in polyunsaturated fatty acids (PUFA), conjugated linoleic acid (CLA) and α-tocopherol [6,7]. Some authors argue that the higher the level of grazing, the more phenolic compounds, fat-soluble vitamins, bioactive lipid components, monounsaturated fatty acids (MUFA), PUFA and CLA are present in the milk [3,8].

This variation in the analytical characteristics of milk depending on the management system adopted by the farmer has been extensively studied in the cattle sector, as in the study developed by Butler et al. [9,10], who concluded that milk obtained under extensive systems had higher fat and protein contents (45 g/kg vs. 41 g/kg for fat and 35 g/kg vs. 33 g/kg for protein) and a higher ω3/ω6 ratio (0.42 vs. 0.30). Regarding goats, several studies assert that farms with a medium-high level of grazing produced higher quality milks since they had higher values of MUFA, PUFA, α-tocopherol and omega 3 fatty acids (ω3) [7,11]. However, concerning sheep milk, studies are much more scarce. Revilla et al. [12] showed that milk fat, protein and lactose contents were not significantly influenced by the management system (conventional vs. organic), although organic milk had significantly higher values of MUFA, PUFA and CLA and lower values of saturated fatty acids (SFA).

In the region of Castilla y León, conventional management systems in the dairy sheep sector can be grouped according to a criterion that considers the grazing level [13]: intensive farms, where sheep are continuously stabled and their feed is based on the forage and concentrate supplemented by the farmer; semi-intensive and semi-extensive farms, where a limited level of grazing is combined with stabling, and in which the feed is based both on the grass intake of the sheep and on the supplementation of forage and concentrates; and, finally, extensive farms, where sheep obtain all their feed from grazing, although there may be a small input of supplementary feed when necessary.

Nevertheless, it is not only milk quality that varies in each of the management systems, but also the carbon footprint indicators associated with milk production. Currently, there is no clear evidence that extensive production systems are preferable to intensive systems from an environmental point of view in terms of greenhouse gas (GHG) emissions. In fact, some authors claim that extensive farms generate a greater amount of methane from enteric fermentation than intensive farms [14], being one of the main GHG associated with livestock [15,16], but there are also detractors to this argument [17]. Many studies have shown that, overall, extensive systems have a lower environmental impact than intensive systems because they use their own inputs instead of external ones, thereby reducing their energy consumption and, consequently, the fossil fuels they require [18,19,20].

Considering the above, the starting hypothesis proposed in this study is that the level of grazing will determine both the milk quality and the farm’s carbon footprint indicators. It is also intended that, with these quality and carbon footprint indicators, it is possible to establish a farm clustering that will provide additional information on the management system of the farms. Therefore, the aim of this study is to assess the grazing level influence on milk quality and carbon footprint indicators and use them to identify the management system of several dairy sheep farms.

## 2. Materials and Methods

### 2.1. Data Collection

This work was based on a 1-year monthly study of 17 dairy sheep farms located in the region of Castilla y León (northwestern Spain). Among these farms, seven used the local “Churra” breed while 10 used the Assaf breed. During each monthly visit to the farms, data regarding operational aspects, input and output quantities and their economic impact, milk quality and direct and indirect environmental parameters on the management system were collected from sampling, interviews and farm record analysis.

The data collected were used to construct representative indicators for each of the three groups of variables, namely 10 indicators on the farm management system, 11 on milk quality parameters and 5 on environmental parameters related to the carbon footprint (GHG emissions and energy efficiency).

### 2.2. Milk Quality Parameters

The samples were taken from the tank of each of the sheep farms in each monthly sampling. Duplicates of milk samples were placed in 100 mL plastic containers. The aliquots for the analysis were taken from one of the 100 mL samples, while the other 100 mL sample was preserved for analysis if needed. A total of 204 samples were analysed.

Fat, protein and dry extract were analysed using a Milko Scan (Foss Analytical, Hillerød, Denmark). Lipids were extracted using the International Standard Method ISO 14156:2001 [21]. Briefly, 25 mL of milk, 20 mL of 96% ethanol, 5 mL of NH_3_ 14 M and 25 mL of ethyl ether were added into a separating funnel and shaken. After settling, 25 mL of 99% n-pentane was added and shaken. It remained standing until phase separation, then the aqueous phase was removed. Over the organic phase, 25 mL of Na_2_SO_4_ 10% was added and the aqueous phase was removed after shaking. Solid Na_2_SO_4_ was added and filtered. The organic solvent was removed at a rotary evaporator to obtain the extracted fat.

Extracted fat (0.1 g) was methylated with KOH 0.2 M in anhydrous methanol by heating at 50 °C for 30 min. After cooling, methylated fatty acids were extracted with 2 mL of hexane. The organic layer was separated and analysed according to the method of Lurueña-Martínez et al. [22] using a gas chromatograph GC 6890 N (Agilent Technologies, Santa Clara, CA, USA) equipped with a 100 m × 0.25 mm × 0.20 µm capillary column (SP-2560, Supelco, Inc., Bellefonte, PA, USA) and an FID detector. The conditions of the oven temperature were as follows: the initial temperature was 150 °C and it increased at 1 °C/min until 165 °C, afterwards an increase at 0.20 °C/min up to 167 °C was programmed and finally it increased by 1.50 °C/min up to 225 °C. This temperature was held for 15 min. The injector and detector temperatures were 250 °C. Then, 1 μL of the extracted fatty acids was injected into the chromatograph in split mode (20:1). The carrier gas was helium at 1 mL/min. The fatty acids, including CLA isomers, were identified by their retention times, using a mixture of 37 fatty acid standards (47885-U Supelco, Sigma-Aldrich, Steinheim, Germany) and four CLA isomers (Larodan Fine Chemicals AB, Malmo, Sweden) and their contents were calculated using chromatogram peak areas and expressed as g per 100 g total fatty acid methyl esters.

To analyse the tocopherol isomers, the vitamins were extracted according to the method proposed by Herrero-Barbudo et al. [23] and briefly modified by Gutiérrez-Peña et al. [11]. Then, samples were heated (30 °C) and subjected to alkaline hydrolysis after homogenization. Subsequently, 1.5 mL of ascorbic acid 0.3 M and 0.1 mL of δ-tocopherol as internal standard were added to 2 mL of sample. The mixture was vortexed and methanolic KOH (40%) was added and the mixture was then vortexed again for 30 s. The mixture was heated to 70 °C and shaken (200 rpm) for 40 min. Samples were cooled for 3 min and extracted using n-hexane:dichloromethane (5:1)/isopropanol (4/1) four times. The organic phases were pooled, washed with cooled water to remove KOH, evaporated under a nitrogen flow, reconstituted in 1 mL of acetonitrile/methanol (85/15) and filtered using a 22 μm syringe filter.

Chromatographic analysis was performed according to Chaveau-Duriot et al. [24] with the minor modification proposed by Gutiérrez-Peña et al. [11]. UPLC was run on a Waters Acquity system (Waters, Guyancourt, France) equipped with a photodiode array detector, scanning at between 275 and 465 nm, and a fluorometric detector. A 150 × 2.1 mm^2^ Acquity UPLC HSS T3, 1.8 mm column (Waters, Guyancourt, France) was used. The flow rate applied was 0.4 mL/min and the analyses were performed at 35 °C [11]. The isocratic method employed used an acetonitrile:methanol (85:15)/isopropanol: water (50:50) in 80/20 proportion mobile phase. The fluorometric detection used a λexc = 295 and λem = 330 nm. Peak identification was accomplished using pure standards (Sigma-Aldrich, Steinheim, Germany) and quantification was performed using calibration curves. The purity of the standards used was monitored by UV-Vis spectra. With this method, it was not possible to separate the α- and β + γ-tocopherol forms and they were computed together.

### 2.3. Carbon Footprint Indicators

The life cycle assessment (LCA) methodology was used to determine the carbon footprint and to assess the environmental impact of the farms. The PAS 2050:2011 specification for life cycle assessment of GHG emissions from goods and services [25] per production unit and the Intergovernmental Panel on Climate Change (IPCC) guidelines for national GHG inventories were followed [26]. This methodology is based on that described by Batalla et al. [27]. The boundary chosen for the ewe milk production system was “from cradle to farm gate”. This range included all the on-farm (livestock enteric fermentation and N_2_O soil management emissions) and off-farm emissions (mainly processing and transport of all the inputs used on the farms). Machinery, buildings, medicines and other minor stable supplies were excluded from the assessment.

The functional unit (FU) used is 1 kg of fat and protein corrected milk (FPCM), which is the most common FU for carbon footprint calculation. Instead of individually assessing each of the carbon footprint indicators in FU terms, carbon footprint final value was calculated using this unit. The equation proposed by Pulina et al. [28] was used to correct fat and protein content. This work is a mass assignment (inputs and outputs quantities) where all emissions were attributed to milk and lamb production.

The study analysed the net energy requirement (MJ/sheep/year) and the levels of CO_2_, CO_2_ eq, CH_4_ and N_2_O emitted (kg/sheep/year). While CH_4_ from enteric fermentation was calculated following Merino et al.’s [29] procedure, the rest of the GHG mentioned were estimated according to IPCC guidelines [26], considering the latest updates [30]. CH_4_ from enteric fermentation was calculated using the following equation:

(1) $$Emissions=EF_{\left(T\right)}\times \left(\frac{N_{\left(T\right)}}{10^{6}}\right)$$

where Emissions: CH_4_ emissions from enteric fermentation (Gg CH_4_/year); $EF_{\left(T\right)}$: emission factor for the defined livestock population (kg CH_4_/sheep/year); $N_{\left(T\right)}$: number of sheep in the farm; T: livestock category.

Direct $N_{2}O$ emissions from managed soils (Tier 1) were calculated as follows:

(2) $$N_{2}O_{Direct}-N=N_{2}O-N_{N\;inputs}+N_{2}O-N_{OS}+N_{2}O-N_{PRP}$$

where $N_{2}O_{Direct}-N$: annual direct $N_{2}O_{Direct}-N$ emissions produced from managed soils (kg N_2_O − N/year); $N_{2}O-N_{N\;inputs}$: annual direct N_2_O − N emissions from N inputs to managed soils (kg N_2_O − N/year); $N_{2}O-N_{OS}$: annual direct N_2_O − N emissions from managed organic soils (kg N_2_O − N/year); $N_{2}O-N_{PRP}$: annual direct N_2_O − N emissions from urine and dung inputs to grazed soils (kg N_2_O − N/year).

According to the latest Synthesis Report (SYR) of the IPCC Fifth Assessment Report (AR5) [31], NO_2_ and CH_4_ Global Warming Potential (GWP) values, i.e., values for cumulative forcing over 100 years, are 28 and 265, respectively.

These GHG emission factors, together with the FU, were used to calculate the carbon footprint using the equation:

(3) $$CF=\frac{\left(CO_{2}\times GWP_{100}\right)+\left(CH_{4}\times GWP_{100}\right)+\left(N_{2}O\times GWP_{100}\right)}{FPCM}$$

where CF is the carbon footprint and $GWP_{100}$ is the global warming potential, cumulative forcing over 100 years [31].

### 2.4. Management System

Management system data were obtained through interviews with farmers and the available farm data records. Therefore, the results of this work are directly applicable to the ewe dairy sector. This information included data on the number of mature sheep on the farm, the milk production individualized per sheep and per year calculated using the official milk recording method approved by the International Committee for Animal Recording (ICAR) [32], the total percentage of forage and concentrate included in the diet, the percentage of total forage that came from green pasture, the percentage of grazing and stabling time, the useful agrarian land (UAL) measured in hectares (ha), the negative energy balance (NEB) measured in megajoules (MJ) and the net margin or economic income expressed in EUR per sheep. The main characteristics of each of the farms are shown in Table 1.

All indicators were calculated similarly as Mena et al. [33]. The indicator “sheep per farm” refers to all ewes included in the breeding group, obtaining the annual value as the average of ewes present each month. The indicators “total forage” and “total concentrate” were obtained by weighing the amount of forage and concentrate provided to the flock, expressed as a percentage of the total diet. The indicator “pasture in total forage” was calculated as the part of the forage intake, which is provided by the pastures, expressed as a percentage of the total forage intake. The indicators “grazing time” and “stabling time” were calculated by counting the daily hours that the flock was grazing or stabled, expressed as a daily percentage, averaging the annual value from those daily results. The “milk production” indicator was calculated as the sum of the daily milk production of the flock in the study year divided by the indicator “sheep per farm”. The “NEB” indicator was calculated as the difference between the energy outputs (calorific value of milk, lambs and crops produced in the farm) and the energy inputs (directly consumed are fuels and electric energy; indirectly consumed are energy used for elaboration, manufacturing of products and transport of outputs). The economic profitability indicator “net margin” was calculated as the difference between farm incomes (i.e., milk and lamb sales) and farm costs (i.e., feeding costs such as expenditure on concentrate, forages, crops and land rent).

### 2.5. Statistical Analysis

Statistical processing of the data was carried out using IBM-SPSS Statistics 26 software (IBM, Chicago, IL, USA).

A factor analysis was carried out using the principal components method with an orthogonal Varimax rotation as a particular solution. The factor scores that summarized the information were saved using the Anderson–Rubin method [34]. Kaiser–Meyer–Olkin (KMO) measure of sampling adequacy [35] and Bartlett’s Test of Sphericity [36] were carried out to verify data suitability for factor analysis.

A cluster analysis was performed using the obtained factor scores, following an agglomerative hierarchical algorithm and applying Ward’s method [37] to maximize intra-cluster homogeneity.

In order to graphically represent, in a two-dimensional space, the separation between the different farmers (and consequently, between the different clusters) in terms of Euclidean distance, a population canonical analysis or discriminant coordinate analysis was carried out. For this purpose, the cluster membership of the farmers was used as a grouping variable.

Significant differences among clusters for each of the milk quality and carbon footprint indicator parameters were obtained by a multivariate analysis of variance (MANOVA) fitted to a generalized linear model (GLM) [38]. Means and standard deviations (SD) were calculated for all variables. The statistical significance of each factor was assessed at a 95% confidence level (α = 0.05) using Snedecor’s F as the contrast statistic. For differentiation of homogeneous subsets, Tukey’s test [39] was used. Regarding the management system parameters, the same process was used to find significant differences between clusters.

## 3. Result and Discussion

### 3.1. Procedure Grounds

Once the data had been collected from all the farms, it was decided to design a statistical procedure aimed at identifying natural groupings between the different farms. As shown in Table 2, although some correlations were statistically significant, Pearson’s linear correlation coefficients showed considerably low absolute values, so it can be assumed that milk and carbon footprint indicators are not strongly correlated enough to be able to draw conclusions from one indicator on the basis of the other. For this reason, it was necessary to use statistical techniques capable of detecting their latent relationships.

A multivariate procedure was chosen because of its potential to detect non-observable relationships between different variables [40]. This procedure was carried out on the grounds of three fundamental techniques. Factorial analysis scores that algebraically gather the most relevant information from the initial variables were used as a starting point. These factor values were then subjected to an agglomerative hierarchical cluster analysis to obtain a dendogram. This dendogram showed the natural clustering among the different farms, resulting from the latent relationships between milk quality and carbon footprint indicators. In view of this dendogram, it was considered that the most appropriate solution or number of clusters would correspond to the one obtained from the stage of the iterative process immediately preceding the stage in which there were abrupt leaps in the distance between clusters [41]. Finally, a canonical population analysis was carried out in order to graphically evidence the results obtained in the cluster analysis in a two-dimensional space.

### 3.2. Factor Analysis

Due to the high number of variables involved in this work, it was deemed necessary to reduce the dimensionality of the data, in order to make sure that only the relevant information contained in the dataset is used. The main results of that factor analysis are shown in Table 3. The data adequacy to this multivariate technique appeared to be satisfactory since the value of the KMO measure was higher than 0.6 and the null hypothesis of Barlett’s sphericity was rejected with a *p*-value of less than 0.05, coinciding with the criteria of Cuadras [42]. The analysis identified six relevant factors that accounted for 85.34% of the variance. Each of the variables contributed to a greater or lesser extent to each of the factors; however, the greater contribution of a variable to a specific factor is what will determine the result of the subsequent analyses, i.e., the cluster and the canonical population analysis. The parameters fat content, protein content, dry extract and α-tocopherol were the most important contributors to factor 1; SFA, MUFA, PUFA, CLA and ω6 to factor 2; ω3 and β+γ-tocopherol to factor 3; CO_2_ and CO_2_eq to factor 4; energy consumed and N_2_O to factor 5; and only CH_4_ to factor 6.

Factor 1 is thereby related to milk’s basic composition and α-tocopherol, factor 2 is associated with milk’s lipid profile, showing the saturated (SFA) and unsaturated fatty acids (MUFA, PUFA and CLA) have an inverse correlation, and factor 3 is linked to green pasture as this increases the ω3 content, although it showed a negative correlation with the β+γ-tocopherol content. These three factors are consequently associated with milk composition. On the other hand, factor 4 is linked to the farm’s carbon footprint and factors 5 and 6 to the energy needed for operation and other GHG emissions.

### 3.3. Cluster Analysis

**Figure 1:**
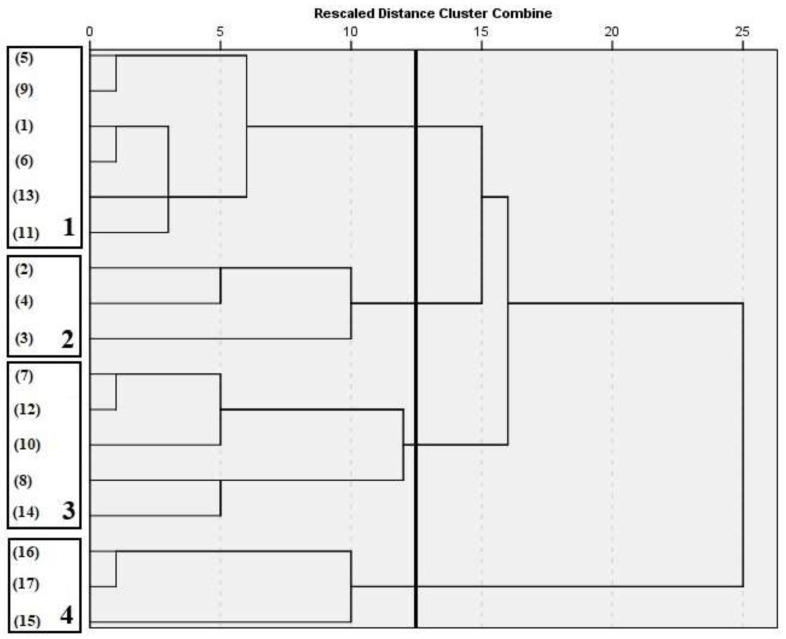
Dendrogram obtained from hierarchical cluster analysis with Ward’s method. On the left, the farms that make up each of the clusters are grouped together. Each number (1–4) corresponds to each of the formed clusters. Cut-off point: 12.5.

Based on the factor scores of all the parameters studied in each of the factors generated by the statistic test, a cluster analysis was carried out to detect natural clustering between farms. Figure 1 shows the dendrogram resulting from that cluster analysis (*N* = 17 farmers), from which it was determined that 12.5 was the ideal cut-off distance, since it is the point just before the iterative process showed big gaps between subsequent cluster’s distances.

Cluster 1 was formed by six farmers (numbers 1, 5, 6, 9, 11 and 13), cluster 2 by three farmers (numbers 2, 3 and 4), cluster 3 by five farmers (numbers 7, 8, 10, 12 and 14) and cluster 4 by three farmers (numbers 15, 16 and 17). Each of these clusters presented different characteristics in terms of management system (Table 4), milk quality (Table 5) and carbon footprint indicators (Table 6). Therefore, fitting the results derived from the management systems analysis of the different clusters to the classification established by De Rancourt et al. [13], cluster 4 would belong to the “extensive” class, cluster 3 to the “semi-extensive” class, cluster 1 to the “semi-intensive” class and cluster 2 to the “intensive” class.

### 3.4. Canonical Population Analysis

**Figure 2:**
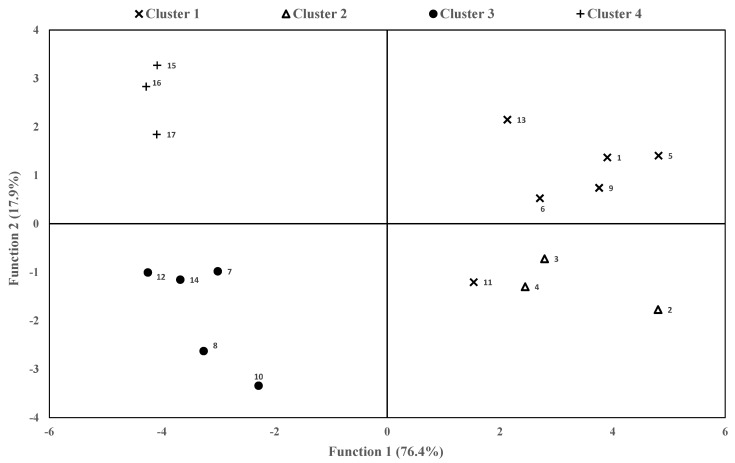
Scatter plot that shows the canonical or discriminant coordinates of the studied farms. In brackets is the % variance that explains each of the canonical functions. Numbers near the symbols identify the position of the different farms.

All indicators (milk quality and environmental) were reduced to two canonical functions that together cover 94.3% of the total variance. Figure 2 shows the scatter plot whose axes are represented by these two canonical functions and where the 17 farms are positioned in a bidimensional space. This diagram shows the clear formation of the four different clusters of farms, except for farm 11, which was separated from its cluster. Fat content, α-tocopherol, energy consumed, CO_2_ and CO_2_eq were selected by the analysis as the five parameters with the greatest discriminant power. The first canonical discriminant function revealed a direct relation with energy consumed, CO_2_, N_2_O and CO_2_eq and an inverse relation with protein content, PUFA, CLA, ω3 and α-tocopherol. On the other hand, the second canonical discriminant function showed a direct relation with fat content, dry extract and SFA and an inverse relation with ω6 and PUFA.

Therefore, according to the interpretation of the scatter plot, these preliminary results would suggest that farms in clusters 1 and 2 would have higher energy consumption and higher GHG emissions and their milks would have low PUFA, CLA, ω3 and α-tocopherol content. In contrast, farms in clusters 3 and 4 would have lower energy consumption and environmental impact, and their milks would have higher contents of the above-mentioned parameters. On the other hand, milks from farms in clusters 1 and 4 will have a higher fat content, higher dry extract and higher SFA content, but lower ω6 and PUFA content. Farms in clusters 2 and 3 will have the opposite characteristics.

### 3.5. Cluster’s Management Characteristics

Table 4 shows the mean values of the management characteristics considered for each of the resulting clusters. Cluster 4 had the highest number of ewes per farm, being significantly higher than clusters 1 and 2, which in turn were significantly higher than cluster 3, with the lowest value for this parameter. The average annual milk production per ewe was significantly different in each of the clusters, being higher in cluster 2 (441.7 l/sheep/year), which was 44.8%, 63.0% and 90.1% higher that in clusters 1, 3 and 4, respectively. The total forage percentage varied by 11.7% between the highest value shown by cluster 1 (72.3%) and the lowest value shown by cluster 2, the latter being statistically different from the others. On the other hand, regarding the pasture portion included in the total forage consumed, cluster 4 (46.4%) showed a significantly higher value than the rest of the clusters, being 83.0%, 96.8% and 99.8% higher than that of clusters 3, 1 and 2, respectively. The mean values of concentrate consumed were inversely related to the values of total forage consumption, since cluster 2 (39.4%) had a significantly higher value than the rest of the clusters, while cluster 1 had a significantly lower value, with a variation of 13.8% between the two clusters. Grazing time is inversely related to stabling time, the ewes of cluster 4 (87.1%) being the ones that had a higher grazing level and lower stabling time compared to the rest of the clusters, contrary to what happens with the ewes of clusters 1 and 2, these differences being statistically significant. UAL is directly related to grazing time, cluster 4 (874.3 ha) being the one that showed significantly higher values than the rest of the clusters, namely 90.9%, 94.1% and 95.6% higher than the values of clusters 1, 3 and 2, respectively. NEB is related to grazing and stabling times, being the clusters with higher grazing time and lower stabling time [clusters 3 and 4 (clusters average −1,053,222 MJ)] the ones with significantly higher NEB values. Regarding the net margin per ewe, it was cluster 4 (88.8 EUR/sheep/year) that showed a significantly higher value of this parameter than the rest of the clusters (46.3%, 51.8% and 66.2% more than clusters 2, 1 and 3, respectively), considering that this cluster is the one with more ewes per farm, more grazing time, more UAL and higher NEB, but lower milk production per ewe per year and shorter stabling time. All these results were closely related to ewe breed—cluster 2 consisted exclusively of Assaf breed farms and cluster 4 consisted exclusively of Churra breed farms, whereas clusters 1 and 3 had both types.

### 3.6. Cluster’s Milk Quality

Milk quality characteristics of the four clusters of farms, previously determined by multivariate methods, are shown in Table 5. Statistically significant differences were found for protein content, total fat content and dry extract, obtaining in all of them the highest value in cluster 4 and the lowest in cluster 2. The protein content was directly correlated with the grazing level (pasture in total forage) and total forage and inversely correlated to the milk production (Table 4), showing a Pearson correlation coefficient of 0.50, 0.25 and −0.33 (*p* < 0.01), respectively. Previous works point out that dairy cows grazing *ad libitum* had higher concentrations of milk protein and casein than animals grazing a restricted pasture allowance [43] or with a total mixed ratio [44]. However, these results are more in agreement with previous works that reported lower milk yield and higher protein content in high grazing level ewes [45] and cows [9], while there were no differences in protein content with grazing level when milk yield was unaffected [7] or a higher protein content was observed for feedlots that had lower milk production compared to pasture grazing ewes [46]. These observations can be explained due to a dilution effect [47]. This is due to the fact that at a particular level of energy intake, there is a minimum protein intake, and reduction below this protein level will cause a reduction in milk yield [3].

On the other hand, and regarding the correlation observed with total forage, previous works have shown that there is strong evidence showing an increase in milk protein in dairy cows when the forage:concentrate ratio increases [48], but it depends on the energy density of the diets [2] and their composition.

Regarding fat content (Table 5), usually this component decreases when the amount of ingested concentrate increases [49], as observed for clusters 2 and 3. There was not a significant correlation between milk yield and fat content, because at certain levels, even when the milk yield increases, the fat content does not decrease significantly [50]. Moreover, when the objective is to acquire sheep milk with a higher fat content, the strategy adopted is that of supplying a fat-supplemented diet [51]. That might possibly be reason for the high fat content of cluster 2, in spite of the low forage proportion. Regarding the effect of grazing level, there was a significant correlation (0.161, *p* < 0.05) between fat content and pasture in total forage as previously reported by Sales-Duval et al. [52] and differing from the results found by Delgado-Pertiñez et al. [7] which did not determine a significant effect of grazing level on fat content. A negative energy balance produced by undernutrition in grazing animals will result in an increase in milk fat content due to an increase in free fatty acids in blood, which is a consequence of body fat mobilization [53]. Other authors reported that grains can provide a high proportion of starch for digestion in the small intestine, leading to an increase in milk yield and a decrease in milk fat concentration [54].

Dry extract showed same results as fat content, because protein content is more stable than fat and lactose usually did not show significant variation due to diet [12], and the differences observed are likely linked to the variations in milk production [2]. Besides the effect of management system, the effect of breed should be taken into consideration. Previous works have shown that Churra milk had significantly higher protein, fat and dry extract content than that of the Assaf breed [55,56], which is directly related to clusters 4 and 2, respectively.

No significant differences among the four clusters were found for SFA, MUFA and PUFA (Table 5). Indeed, no significant correlations were observed for these parameters with milk production or grazing level, except for PUFA, which showed a significant correlation (0.210 *p* < 0.01) with the percentage of pasture in total forage. Regarding CLA levels (Table 5), there were no significant differences among the clusters (*p* = 0.070), but Pearson coefficients showed a significant correlation between CLA content and the percentage of pasture in total forage (0.511 *p* < 0.01) and with grazing time (0.323 *p* < 0.01). Similar results have been previously reported for grazing ruminants. Then, organic ewe’s milk had a higher content of PUFA and CLA [12,57]. Indeed, some works revealed that although CLA content tended to increase with the grazing level [8,58], this is not always observed for PUFA [58]. On the other hand, the lack of statistically significant differences among the four clusters could be related to the high variability of CLA contents within each cluster throughout the year, mainly due to the differences in pasture composition [11]. Previous results showed that grazing animals had the highest levels of CLA in ewe milk during spring [59]. A significant correlation between CLA content with both ω3 and ω6 were observed (0.622 and 0.232, respectively, *p* < 0.01) because most CLA isomers originate from microbial hydrogenation in the rumen and subsequent enzymatic desaturation of hydrogenated intermediates in the mammary gland, mainly from α-linolenic and linoleic acid [60], which are the major fatty acids of the total ω3 and ω6.

Finally, ω6 did not show significant differences between the four clusters, but ω3 content was significantly higher in cluster 4 than in cluster 2 (Table 5). The ω3 showed a direct correlation with % of pasture in total forage (0.498 *p* < 0.01) and time in pasture (0.561 *p* < 0.01), as previously observed for CLA, while the ω6 content showed an inverse correlation with these two variables (−0.239 and −0.410, *p* < 0.01). This means that the grazing level produced a linear increase in ω3 and a linear decrease in ω6 in milk, as was also previously reported by Couvreur [58], because the hydrogenation level was similar between diets. The increase in ω3 is due to the higher concentration of these fatty acids, mainly α-linolenic acid, in fresh pasture [61]. However, not only the amount of pasture, but also the forage species and its phenological phase had a strong influence on fatty acid composition [62,63]. On the other hand, this study showed that ω6 content depended on concentrates of the diet that should be the source of these PUFA. Fatty acids containing more than 18 atoms of carbon are inhibitors of the novo fatty acid synthesis. Therefore, the linear increase in these fatty acids induced a decrease in the short- and medium-chain FA contents. As previously observed for fat, protein, dry extract and ω3, the content of α-tocopherol was significantly higher in cluster 4, while cluster 2 showed the lowest value (Table 5). The Pearson correlation coefficients showed a direct correlation with % of pasture in total forage and grazing time (0.491 and 0.514, respectively, *p* < 0.01), as observed for the above parameters. The correlation between grazing level and α-tocopherol was previously reported [11,64]. This is linked to the fact that fat-soluble vitamins secreted into ruminant milk depend directly on their level in the ration [65]. Therefore, the high α-tocopherol content of green pasture results in a higher transference from blood into milk [11]. Regarding β+ γ-tocopherol, no significant differences were observed among clusters, as previously reported by Gutiérrez-Peña et al. [11], and no significant correlation with grazing level was observed. A significant correlation with α-tocopherol (Pearson’s coefficient value of 0.207, *p* < 0.01) was observed because this is the main active form of vitamin E and is selectively incorporated among eight isomers that are naturally found in plants [66].

### 3.7. Cluster’s Carbon Footprint Indicators

Table 6 shows the carbon footprint indicators for the different farm clusters. The energy consumed was significantly higher in cluster 1 in contrast to cluster 4, which showed the lowest value; the latter coincided with the cluster of more extensive farms where concentrate intake is lower and higher in forage and pasture (Table 4). This is consistent with other studies [67] where the animals that were fed a high level of concentrate had a higher energy consumption. According to Eldesouky et al. [68], higher intensive systems have a negative impact on the environment due to greater energy requirements of livestock, as well as higher pollutant emissions, mainly from the transport of raw materials. The CH_4_ levels did not show significant differences among the four described clusters. This is consistent with the results of other works [15,16], in which the two main emissions contributing to the carbon footprint on farms come from enteric emission and feed. The methane concerned is released as a product of enteric fermentation in the rumen, which depends on the ruminant’s own nature and not on the use of other fossil sources, which are indeed affected by the management system [68]. CO_2_ emissions were significantly lower in cluster 4, while the highest value was found in cluster 2, a result that is inversely related to grazing time. Concerning N_2_O emissions, these were significantly higher in cluster 1, showing the lowest value in cluster 4. CO_2_ and N_2_O, together with CH_4_, are the main GHG emissions [69]. CO_2_ and N_2_O are mainly produced during fossil fuel combustion, coinciding with the highest values in the clusters where the level of external inputs is the greatest [16], i.e., clusters 1 and 2. CO_2_eq was significantly higher in cluster 2, in contrast to cluster 4, which had the lowest value.

The lowest carbon footprint indicators values were found on farms with a higher grazing level and higher natural resource consumption instead of external inputs, associating this type of farms with a significant reduction of GHG emissions [17]. This is consistent with other studies where dairy production from pasture-fed animals was associated with a reduction in GHG emissions [14]. Conversely, other authors claim that intensive farms produce lower GHG emissions than extensive farms [70]. Regarding the carbon footprint, semi-intensive dairy farms with stabled animals had a significantly lower carbon footprint than semi-extensive dairy farms with free-grazing animals. However, considering soil carbon storage (SCS) in the carbon footprint calculations, SCS is higher on farms with higher grazing levels, consequently reducing their GHG emissions [27]. This aspect is further supported by the work of Gutiérrez-Peña et al. [71], where SCS was lower on intensive farms than on extensive farms.

When carbon footprint total emissions are expressed as kg of FPCM, milk production is a very influential factor, as more productive systems reduce carbon footprint. This is exactly what happened in this study, where farms belonging to clusters 3 and 4 showed significantly higher total emissions values than farms from clusters 1 and 2. This is consistent with the results found by Robertson et al. [72] in dairy goat farms in New Zealand, where grazing goat farms had a significantly higher carbon footprint per kg of FPCM compared to intensive farms.

## 4. Conclusions

By using multivariate statistical techniques on milk quality parameters and carbon footprint indicators of dairy sheep farms, which are initially not correlated, it is possible to identify the different management systems of those farms according to the use of natural resources by grazing animals. Subsequently, farms can be grouped on the basis of their management system so that their environmental impact and their milk quality can be extracted from that grouping. For this purpose, the indicators with the highest discriminating power were fat content, α-tocopherol, energy consumed, CO_2_ and CO_2_eq.

Farms contained in cluster 4 (more extensive) used mainly natural grazing resources, while the concentrate purchase and energy consumed were very low. Among the farm clusters, this one showed the lowest environmental impact because of their low GHG emissions and NEB. Their milk showed high PUFA, CLA, ω3 and α-tocopherol levels, i.e., higher content of healthier fatty acids and vitamins.

On the other hand, farms from cluster 2 (more intensive) showed a very high external input dependence. Therefore, their energy consumed and carbon footprints were notably higher than those of cluster 4. Regarding their milk quality, healthy fatty acid and vitamin content were much lower than in cluster 4 milk.

The higher the use of natural resources, the lower the farm’s required external inputs, gases produced and energy costs.

## Figures and Tables

**Table 1:** Management system characteristics for each of the farms involved in this study.

Farm	Breed	Sheep Per Farm	Milk Production (l/Sheep/Year)	Total Forage (%)	Total Concentrate (%)	Grazing Time (%)	Stabling Time (%)
1	Assaf	774	417	52.95	47.05	0.00	100.00
2	Assaf	1000	373	40.80	59.20	0.00	100.00
3	Assaf	600	417	64.90	35.09	0.00	100.00
4	Assaf	743	535	75.97	24.02	10.00	90.00
5	Assaf	699	185	78.94	21.05	0.00	100.00
6	Assaf	1200	295	68.26	31.74	0.00	100.00
7	Assaf	675	226	59.15	40.84	32.00	68.00
8	Assaf	452	101	68.41	31.59	5.00	95.00
9	Assaf	625	283	82.65	17.34	25.00	75.00
10	Assaf	574	265	29.08	70.91	23.00	73.00
11	Churra	1040	174	60.67	27.23	0.00	100.00
12	Churra	920	121	86.15	13.84	43.00	57.00
13	Churra	280	100	92.63	7.37	32.00	68.00
14	Churra	474	108	75.04	24.96	44.00	56.00
15	Churra	1300	24	81.82	18.17	98.64	11.36
16	Churra	1000	53	51.17	48.82	96.80	13.20
17	Churra	476	54	78.86	21.13	66.00	44.00

Total forage (%) and total concentrate (%) from total diet (%), grazing time (%) and stabling time (%) from total animal time (%).

**Table 2:** Correlations between milk quality and carbon footprint indicators (Pearson’s linear correlation coefficient), n = 204.

Parameter	CO_2_	CH_4_	N_2_O	CO_2_eq	EC
F (%)	0.02	0.10	0.04	0.12	−0.13
P (%)	0.05	0.46 **	0.25 **	0.49 **	−0.31 **
DE (%)	0.06	0.30 **	0.16 *	0.32 **	−0.18 *
SFA (%)	0.05	−0.14 *	−0.05	−0.12	0.05
MUFA (%)	−0.02	0.07	0.04	0.05	0.00
PUFA (%)	−0.10	0.34 **	0.12	0.31 **	−0.29 **
CLA (%)	−0.08	0.53 **	0.21 **	0.55 **	−0.40 **
ω3 (%)	−0.04	0.35 **	0.14 *	0.43 **	−0.36 **
ω6 (%)	−0.07	0.02	−0.01	−0.08	0.02
α-Tocopherol (μg/100 g)	−0.08	0.28 **	0.04	0.37 **	−0.32 **
β+γ-Tocopherol (μg/100 g)	0.04	0.02	0.05	−0.04	−0.01

F: fat content, P: protein content, DE: dry extract, SFA: saturated fatty acids, MUFA: monounsaturated fatty acids, PUFA: polyunsaturated fatty acids, CLA: conjugated linoleic acid, ω3: omega-3 fatty acids, ω6: omega-6 fatty acids, EC: energy consumed. * Correlation is significant at the 0.05 level (2-tailed). ** Correlation is significant at the 0.01 level (2-tailed).

**Table 3:** Higher factor coefficients for each variable. They indicate the strongest relationship between a variable and a factor, i.e., they determine which factor contained more relevant information about a specific variable.

Parameter	Factor 1	Factor 2	Factor 3	Factor 4	Factor 5	Factor 6
Fat content (%)	0.718					
Protein content (%)	0.869					
Dry extract (%)	0.941					
SFA (%)		−0.915				
MUFA (%)		0.704				
PUFA (%)		0.813				
CLA (%)		0.678				
ω3 (%)			0.827			
ω6 (%)		0.649				
α-tocopherol (μg/100 g)	0.861					
β+γ-tocopherol (μg/100 g)			−0.847			
Energy consumed (MJ/sheep/year)					0.854	
CO_2_ (kg/sheep/year)				0.952		
CH_4_ (kg/sheep/year)						0.987
N_2_O (kg/sheep/year)					0.901	
CO_2_eq (kg/sheep/year)				0.762		
KMO = 0.698 BTS = 0.000 Variance = 85.34%						

KMO: Kaiser–Meyer–Olkin measure of sampling adequacy, BTS: Barlett’s Test of Sphericity, SFA: saturated fatty acids, MUFA: monounsaturated fatty acids, PUFA: polyunsaturated fatty acids, CLA: conjugated linoleic acid.

**Table 4:** Management system characteristics of the different clusters of farms (mean ± SD).

Parameter	Cluster 1 (*n* = 6)	Cluster 2 (*n* = 3)	Cluster 3 (*n* = 5)	Cluster 4 (*n* = 3)	Total (*N* = 17)	Significance
Sheep per farm	778 ± 295 ^b^	781 ± 168 ^b^	622 ^c^ ± 183 ^c^	925 ± 345 ^a^	760 ± 276	0.000
Milk production (l/sheep/year)	243.7 ± 103.2 ^b^	441.7 ± 69.3 ^a^	163.6 ± 67.8 ^c^	43.7 ± 14.1 ^d^	220.1 ± 146.7	0.000
Total forage (%)	72.3 ± 13.5 ^a^	60.6 ± 14.9 ^c^	64.0 ± 19.3 ^b,c^	70.6 ± 14.0 ^a,b^	67.5 ± 16.3	0.000
Pasture in total forage (%)	1.5 ± 2.3 ^b^	0.1 ± 0.1 ^b^	7.9 ± 6.7 ^b^	46.4 ± 35.3 ^a^	11.2 ± 22.7	0.000
Total concentrate (%)	25.6 ± 12.4 ^c^	39.4 ± 14.9 ^a^	36.0 ± 19.3 ^a,b^	29.4 ± 14.0 ^b,c^	31.8 ± 16.3	0.000
Grazing time (%)	9.0 ± 13.5 ^c^	3.3 ± 4.8 ^c^	29.9 ± 14.4 ^b^	87.1 ± 15.2 ^a^	28.1 ± 32.2	0.000
Stabling time (%)	91.0 ± 13.5 ^a^	96.7 ± 4.8 ^a^	70.1 ± 14.4 ^b^	12.9 ± 15.2 ^c^	71.9 ± 32.2	0.000
UAL (ha)	79.4 ± 62.0 ^b^	38.3 ± 11.5 ^b^	51.8 ± 34.4 ^b^	874.3 ± 885.2 ^a^	206.9 ± 487.7	0.000
NEB (MJ)	−4,301,705 ± 1,662,001 ^b^	−4,681,787 ± 3,053,319 ^b^	−1,210,717 ± 334,462 ^a^	−895,728 ± 47,008 ^a^	−2,860,497 ± 2,335,021	0.000
Net margin (EUR/sheep/year)	42.8 ± 75.6 ^b^	47.7 ± 127.8 ^a,b^	30.0 ± 42.6 ^b^	88.8 ± 97.8 ^a^	48.2 ± 86.4	0.008

^a. b. c. d.^ Different letters mean statistically significant differences *p* < 0.05. SD: standard deviation, UAL: useful agricultural land, NEB: negative energy balance.

**Table 5:** Milk quality characteristics of the different cluster of farms (mean ± SD).

Parameter	Cluster 1 (*n* = 6)	Cluster 2 (*n* = 3)	Cluster 3 (*n* = 5)	Cluster 4 (*n* = 3)	Total (*N* = 17)	Significance
Fat content (%)	7.00 ± 0.22 ^a,b^	6.75 ± 0.20 ^b^	6.77 ± 0.29 ^b^	7.33 ± 0.25 ^a^	6.94 ± 0.30	0.032
Protein content (%)	5.47 ± 0.18 ^a,b^	5.32 ± 0.14 ^b^	5.59 ± 0.17 ^a,b^	6.00 ± 0.55 ^a^	5.57 ± 0.33	0.040
Dry extract (%)	18.12 ± 0.17 ^a,b^	17.82 ± 0.29 ^b^	17.92 ± 0.32 ^b^	18.91 ± 0.80 ^a^	18.15 ± 0.52	0.017
SFA (%)	71.46 ± 0.96	70.19 ± 1.26	70.35 ± 2.02	71.32 ± 1.67	70.88 ± 1.48	0.522
MUFA (%)	23.69 ± 1.17	25.22 ± 1.50	24.57 ± 1.34	23.40 ± 1.05	24.17 ± 1.32	0.258
PUFA (%)	4.59 ± 0.52	4.58 ± 0.39	5.00 ± 0.73	4.81 ± 0.79	4.75 ± 0.59	0.709
CLA (%)	0.54 ± 0.06	0.56 ± 0.04	0.72 ± 0.11	0.84 ± 0.34	0.65 ± 0.18	0.070
ω3 (%)	0.68 ± 0.11 ^a,b^	0.50 ± 0.21 ^b^	0.81 ± 0.22 ^a,b^	1.01 ± 0.21 ^a^	0.74 ± 0.23	0.026
ω6 (%)	3.012 ± 0.475	3.15 ± 0.17	3.04 ± 0.63	2.53 ± 0.20	2.96 ± 0.47	0.395
α-tocopherol (μg/100 g)	71.38 ± 35.60 ^c^	64.29 ± 28.50 ^c^	91.24 ± 45.40 ^b^	185.03 ± 44.70 ^a^	103.09 ± 53.84	0.011
β+γ-tocopherol (μg/100 g)	10.79 ± 3.96	13.17 ± 5.77	8.30 ± 1.80	11.34 ± 2.35	10.57 ± 3.68	0.337

^a. b. c.^ Different letters mean statistically significant differences *p* < 0.05. SD: standard deviation, SFA: saturated fatty acids, MUFA: monounsaturated fatty acids, PUFA: polyunsaturated fatty acids, CLA: conjugated linoleic acid (sum of the 9c,11t-CLA + 10t,12c-CLA + 9c,11c-CLA + 9t,11c-CLA isomers), ω3: omega-3 fatty acids, ω6: omega-6 fatty acids.

**Table 6:** Carbon footprint indicators and carbon footprint value of the different cluster of farms (mean ± SD).

Parameter	Cluster 1 (*n* = 6)	Cluster 2 (*n* = 3)	Cluster 3 (*n* = 5)	Cluster 4 (*n* = 3)	Total (*N* = 17)	Significance
Energy consumed (MJ/sheep/year)	7711.91 ± 2741.27 ^a^	7164.57 ± 1963.30 ^a,b^	3082.79 ± 977.44 ^b,c^	1545.15 ± 873.29 ^c^	5165.56 ± 3187.20	0.001
CO_2_ (kg/sheep/year)	294.60 ± 141.52 ^b^	578.29 ± 119.50 ^a^	250.61 ± 118.97 ^b^	63.84 ± 23.69 ^c^	291.01 ± 193.18	0.001
CH_4_ (kg/sheep/year)	2.32 ± 0.35	2.37 ± 0.32	2.27 ± 0.39	2.38 ± 0.41	2.32 ± 0.47	0.159
N_2_O (kg/sheep/year)	0.46 ± 0.07 ^a^	0.36 ± 0.02 ^a,b^	0.29 ± 0.06 ^b,c^	0.18 ± 0.03 ^c^	0.34 ± 0.11	0.000
CO_2_eq (kg/sheep/year)	768.12 ± 103.56 ^b^	925.76 ± 122.10 ^a^	597.60 ± 102,50 ^b^	352.11 ± 196.23 ^c^	672.32 ± 205.34	0.000
Carbon footprint (kgCO_2_eq/kgFPCM)	2.45 ± 2.13 ^b^	1.73 ± 0.38 ^b^	3.55 ± 1.37 ^a,b^	6.58 ± 2.78 ^a^	3.37 ± 2.37	0.028

^a. b. c.^ Different letters mean statistically significant differences *p* < 0.05. SD: standard deviation.

## Data Availability

The data presented in this study are available on request from the corresponding author.
